# The Role of DDT in Malaria Control

**DOI:** 10.1289/ehp.1002279

**Published:** 2010-07

**Authors:** Hans Rudolf Herren, Charles Mbogo

**Affiliations:** Millennium Institute, Arlington, Virginia, E-mail: hansrherren@mac.com; *icipe*–International Center of Insect, Physiology and Ecology, Nairobi, Kenya

The letter “DDT and Malaria Control” (Tren and Roberts 2010) is the latest in a long string of opinion pieces placed by authors associated with Africa Fighting Malaria (AFM). Appearing in both the popular media (e.g., AFM 2006; Bate 2009; Bate and De Lorenzo 2007; Roberts 2007a; Tren 2002) and scientific literature (e.g., Attaran et al. 2000; Roberts 2001, 2007b; Roberts et al. 2000, 2004; Tren 2009), these articles and letters reduce the complex issue of malaria control to a single, dichotomous choice between DDT and malaria. Framing the issue in this manner is a dangerous oversimplification and an distraction from the critical dialog on how to effectively combat malaria around the world—particularly in African communities.

The question that AFM and malaria control experts must ask is not “Which is worse, malaria or DDT?” but rather “What are the best tools to deploy for malaria control in a given situation, taking into account the on-the-ground challenges and needs, efficacy, cost, and collateral effects—both positive and negative—to human health and the environment, as well as the uncertainties associated with all these considerations?”

Tren and Roberts (2010) briefly acknowledged that alternatives to DDT exist (while denigrating them as “supposed solutions”), but in typical fashion they focused most of their letter on the chemical, arguing that the health effects of malaria are much worse that those of DDT exposure. As malaria professionals we are well aware of the dire health consequences of malaria, but also of DDT. The challenge before us is therefore to determine how much weight to give to vector control within the broader context of a malaria control program; within vector control, how much weight to allot to nets versus indoor residual spraying (IRS); and within IRS, how much weight to give to DDT or some other chemical.

These decisions are indeed complex and location specific. In this regard, van den Berg’s commentary, “Global Status of DDT and Its Alternatives for Use in Vector Control to Prevent Disease” (van den Berg 2009), is a most useful contribution. In contrast, Tren and Roberts’ (2010) advice that “van den Berg’s concerns should be ignored” strikes us as reckless and irresponsible.

In 2006, Allan Schapira, former coordinator of vector control and prevention of World Health Organization’s Global Malaria Programme, observed that malaria control discussions had become “polluted,” and warned, “The renewed interest in indoor residual spraying could lead to interminable debates in countries about the pros and cons of DDT” (Schapira 2006). However well intentioned, Tren and Roberts (2010)—as with much of AFM’s output—do more to fuel those “interminable debates” than to meaningfully inform decisions that will save people’s lives.

## References

[b1-ehp.118-a282b] Africa Fighting Malaria (2006). Africa Fighting Malaria Responds To Berkeley University Study Into DDT And Neurodevelopment In Children. MedicalNewsToday.com.

[b2-ehp.118-a282b] Attaran A, Roberts DR, Curtis CF, Kilama WL (2000). Balancing risks on the backs of the poor. Nat Med.

[b3-ehp.118-a282b] Bate R (2009). Comment: UN disarms Weapon of Malarial Destruction. Joy Online (Ghana).

[b4-ehp.118-a282b] Bate R, De Lorenzo M (2007). Reconsider DDT against Malaria. New Times (Rwanda).

[b5-ehp.118-a282b] Roberts DR (2001). DDT risk assessments [Letter]. Environ Health Perspect.

[b6-ehp.118-a282b] Roberts DR (2007a). A New Home for DDT. New York Times.

[b7-ehp.118-a282b] Roberts DR (2007b). Preventing malaria in endemic areas [Editorial]. BMJ.

[b8-ehp.118-a282b] Roberts D, Curtis C, Tren R, Sharp B, Shiff C, Bate R (2004). Malaria control and public health [Letter]. Emerg Infect Dis.

[b9-ehp.118-a282b] Roberts DR, Manguin S, Mouchet J (2000). DDT house spraying and re-emerging malaria. Lancet.

[b10-ehp.118-a282b] Schapira A (2006). DDT: a polluted debate in malaria control [Letter]. Lancet.

[b11-ehp.118-a282b] Tren R (2002). Africa needs DDT [Letter]. New York Times.

[b12-ehp.118-a282b] Tren R (2009). Insecticides for public health. CMAJ.

[b13-ehp.118-a282b] Tren R, Roberts D (2010). DDT and malaria prevention [Letter]. Environ Health Perspect.

[b14-ehp.118-a282b] van den Berg H (2009). Global status of DDT and its alternatives for use in vector control to prevent disease. Environ Health Perspect.

